# Severe wound traction-blisters after inadequate dressing application following laparoscopic cholecystectomy: case report of a preventable complication

**DOI:** 10.1186/1754-9493-5-4

**Published:** 2011-03-28

**Authors:** Abayomi L Sanusi

**Affiliations:** 1 Department of Surgery, York Hospital, Wiggington Road, York, North Yorkshire, YO31 8HE, UK

## Abstract

**Background:**

The inadequate application of postoperative dressings can lead to significant complications, including skin injuries, compartment syndromes, and potential limb loss. To our knowledge, the occurrence of post laparoscopic cholecystectomy related skin complications have not yet been reported in the peer-reviewed literature.

**Case Presentation:**

Following laparoscopic cholecystectomy for symptomatic gallstone disease, a seventy eight year old healthy white male broke out in painful erythema on either side of his epigastric port site. Vesicles akin to a partial thickness burns were revealed upon removal of dressings. An unusual indentation created by the dressing, and skin traction by the dressing's adhesive edges were implicated, raising questions about technique of its application.

**Conclusion:**

Incorrect application of wound dressings can disrupt skin architecture, causing painful blistering. This complication should not occur to patients, as it is theoretically 100% preventable. Avoidance of stretching adhesive dressings, and careful adherence to relevant manufacturers' instructions are recommended.

## Introduction

Postoperative peri-wound blistering is a well-recognized phenomenon in Orthopaedic, and to a lesser extent Gynaecological surgery. No cases have previously been reported following Laparoscopic surgery, where incisions are comparatively shorter. Despite a glaring dearth of literature on the topic, there is a recognized association with dressings. We report the first case of post laparoscopic cholecystectomy wound traction blistering, and suggest recommendations to avoid it.

## Case Report

**Figure 1:**
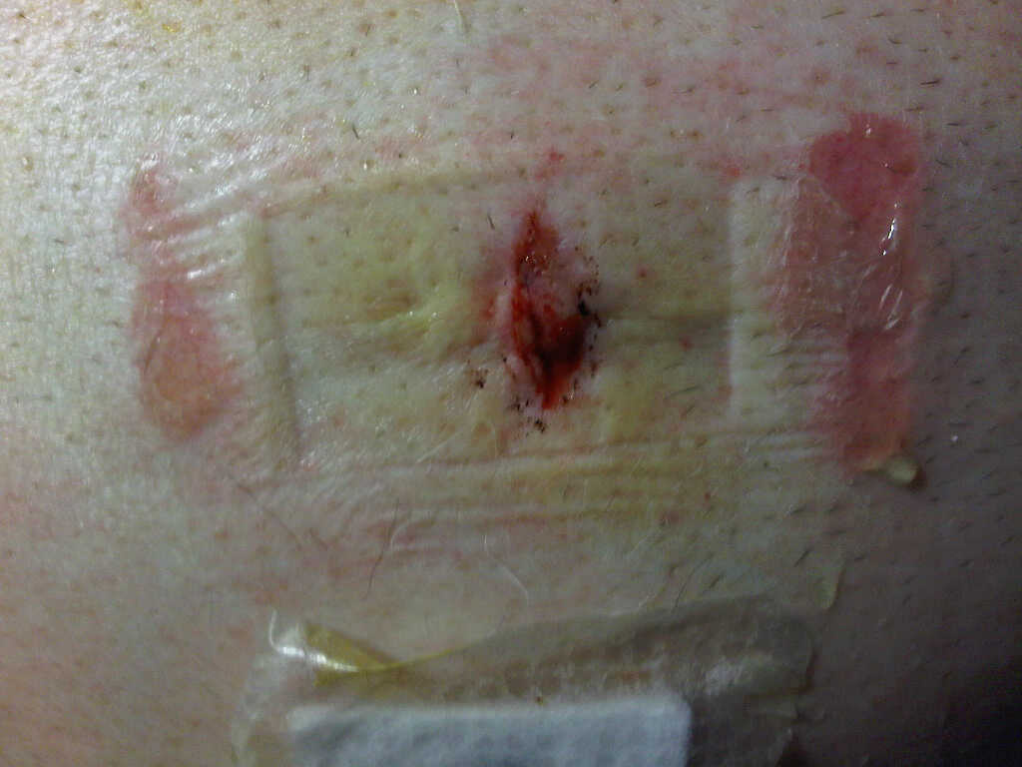
**Painful erythema, blistering and increased local heat around epigastric port incision**. Dressing had been incorrectly applied less than 24 hour earlier.

A 78-year-old healthy white male had an uneventful laparoscopic cholecystectomy for symptomatic gallstone disease. Within 24 hours he developed an area of painful erythema, with blistering and increased local heat on both sides of his epigastric port incision. An area akin to superficial dermal partial thickness burn was in contact with the adhesive lateral aspects of the dressing, where it was fixed to skin [Figure 1].

### Patient's discomfort relieved upon dressing removal

**Figure 2:**
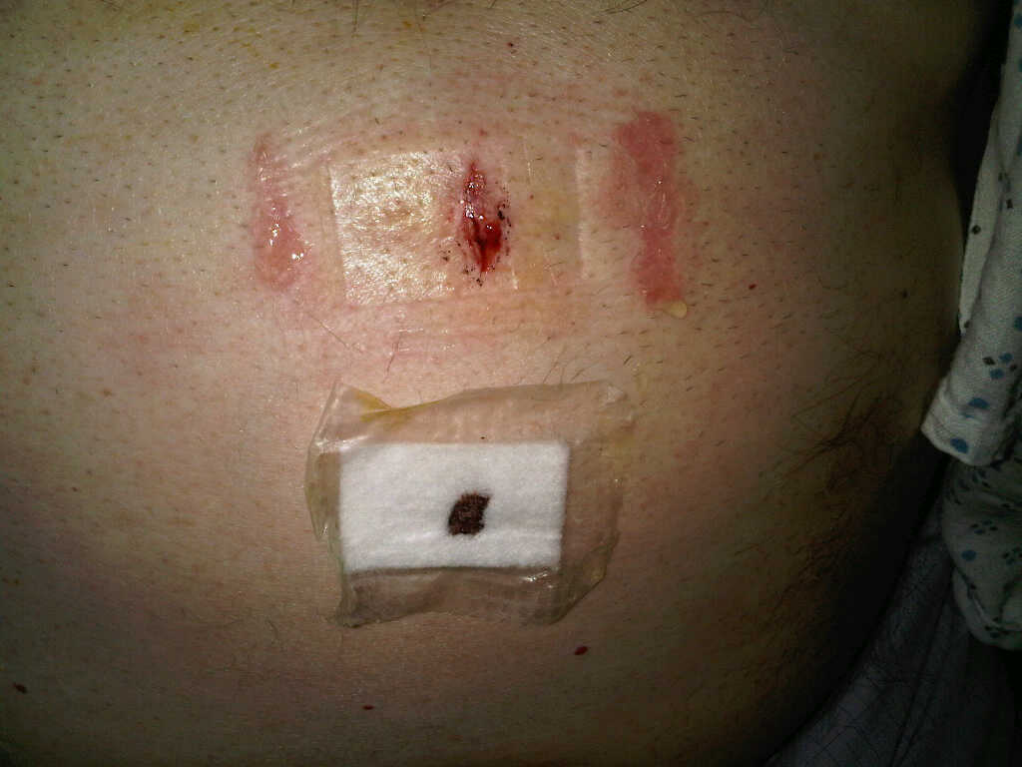
**Well-demarcated indentation on the skin caused by the central rectangular pad of the dressing: this occurred despite no use of pressure dressing**.

**Figure 3:**
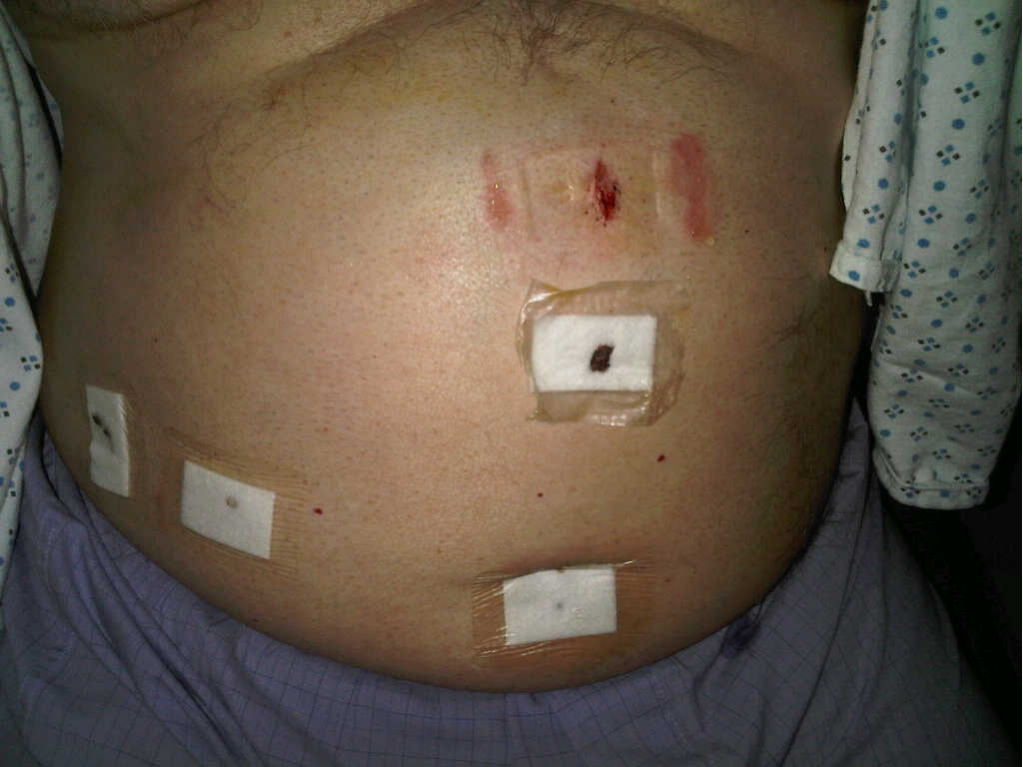
**None of the other three laparoscopic port sites were painful, erythematous, or blistered (normal skin visible through clear adhesive of dressings)**.

It was observed that the central rectangular absorbent-pad part of the dressing had caused a well-demarcated indentation on the skin, although no pressure dressings had been applied [Figure 2]. Retrieval of his gallbladder (using a Bag for Endoscopic Retrieval of Tissue) had been via his umbilical port, which was not affected by the described erythema and blistering. All skin incisions had been made with the scalpel, and there was no use of diathermy on the skin. None of the three other port sites were affected [Figure 3]. The only plausible explanation was the method of application of the patient's dressing. Retrospective assessment of events leading up to onset of pain corroborated incorrect application by stretching of the dressing prior to its application to skin surface.

## Discussion

It is routine practice to apply some form of dressing following elective surgery, since physiologically wound healing is more successful with occlusive dressings [1]. Blistering of skin develops when structural strain is applied to the epidermis, encouraging it to separate from the underlying dermis where it is usually tightly adherent by deep finger shaped projections [2]. There are no previous reports of traction vesicles or dermatitis following laparoscopic procedures, although similar complications are well described in orthopaedic and gynaecological surgery [3]. In orthopaedic surgery postoperative wound blistering is usually due to dressing application, sometimes with tapes, for relatively longer durations [4]. Development of wound blistering increases the risk of surgical site infection, create the need for further dressing application and increase discomfort. It may also increase cost through delayed discharge and outpatient reviews.

There is no previous documented dressing related wound traction blistering, following a laparoscopic procedure. In this context, where the wound length is relatively small compared to incisions in joint replacement or gynaecological surgery, lateral traction by the adhesive surface of the dressing as opposed to friction between skin and dressing, is the implicated mechanism imposing dermal-epidermal separation. Correct and tension free application of dressings even in crusted and untidy epidermal losses [5], as well as tidy incisions will produce lower complication rates and minimize patient discomfort. Manufacturers of occlusive dressings do not recommend stretching prior to application to skin.

## Conclusions

Traction blistering is a cause of morbidity involving laparoscopic access wounds. It is an entirely avoidable complication. Disruption of skin architecture by stretched dressings should be avoided. Persons applying postoperative dressing should do it carefully, and be familiar with specific manufacturer's recommendations.

## Consent

Written informed consent to publish this case report was obtained from the patient.

## Competing interests

The author declares that they have no competing interests.

## Authors' contributions

The author designed and wrote the entire manuscript.
